# Mobile App Integration Into Dialectical Behavior Therapy for Persons With Borderline Personality Disorder: Qualitative and Quantitative Study

**DOI:** 10.2196/14913

**Published:** 2020-06-11

**Authors:** Stephen F Austin, Jens Einar Jansen, Charlotte Juul Petersen, Rasmus Jensen, Erik Simonsen

**Affiliations:** 1 Department of Psychiatric Research, Region Zealand Psychiatry Slagelse Denmark; 2 Mental Health Center Copenhagen Copenhagen Denmark; 3 Psychiatry West, Region Zealand Psychiatry Vordingborg Denmark; 4 Psychiatry West, Region Zealand Psychiatry Holbaek Denmark; 5 Department of Clinical Medicine University of Copenhagen Copenhagen Denmark; 6 Department of Psychiatric Research Region Zealand Psychiatry Slagelse Denmark

**Keywords:** dialectical behavior therapy, mobile application, blended intervention, mHealth, app

## Abstract

**Background:**

The advancement of and access to technology such as smartphones has implications for psychotherapeutic health care and how interventions for a range of mental health disorders are provided.

**Objective:**

The objective of this study was to describe the experiences of participants while using a mobile phone app that was designed to enhance and support dialectical behavior therapy for personality disorders.

**Methods:**

A combination of in-depth interviews and questionnaires were used to capture the experiences of participants who used the app while undergoing dialectical behavior therapy treatment. A mixed methods approach was used; qualitative data from the interviews were analyzed using thematic analysis and were combined with quantitative data from the questionnaires.

**Results:**

Participants (N=24) who were receiving dialectical behavior therapy used the trial app. Participants (n=20) completed an evaluation questionnaire and a subset of this group (n=8) participated in semistructured interviews. Major themes that were identified from the interviews were (1) an overall positive experience of using the app—participants perceived that the app facilitated access and implementation of dialectical behavior therapy strategies (to regulate mood and behavior in challenging situations)—and (2) that the app provided a common source of information for patient and therapist interactions—app-based interactions were perceived to facilitate therapeutic alliance. Qualitative themes from the interviews were largely congruent with the quantitative responses from the questionnaires.

**Conclusions:**

Participants welcomed the integration of technology as a supplement to clinical treatment. The app was perceived to facilitate and support many of the therapeutic techniques associated with dialectical behavior therapy treatment. The incorporation of technology into psychotherapeutic interventions may facilitate the transfer of knowledge and strategies that are learned in therapy to use in real-world settings thereby promoting recovery from mental health problems.

## Introduction

Borderline personality disorder is one of the most prevalent personality disorders in clinical populations [1] and has traditionally been considered difficult to treat [2]. There are a range of structured psychotherapeutic interventions that are considered effective in treating borderline personality disorder [3-5] including dialectical behavior therapy [6,7]. While dialectical behavior therapy is an evidence-based treatment for borderline personality disorder, it has been acknowledged that certain improvements such as reducing dropout rates and improving clinical implementation would benefit treatment effectiveness [8-10].

Advancement of and improved access to technologies such as the internet and mobile apps have led to the examination of how these technologies may supplement and improve the effectiveness of psychotherapeutic interventions [11]. The use of mobile-based interventions can have a number of advantages; mobile-based interventions may facilitate access to evidence-based treatment, enhance the potency of psychotherapy, reduce stigma associated with psychotherapy, enable users to work at their own pace, promote autonomy, and flexibly integrate mental health interventions without local or temporal boundaries into daily life [12-14]. A key component of successful implementation of a mobile app regardless of type of therapeutic intervention is user engagement [15].

Despite a number of potential benefits, there are also a number of concerns such as hacking and the illegal use of personal data, ensuring the quality of the therapeutic interventions that use this technology, the identification and response to adverse events and the impact of technological problems on clinical management and duty of care within vulnerable populations [14,16].

Dialectical behavior therapy is concerned with helping people to increase their emotional and cognitive control through a variety of techniques such as monitoring one’s mood and implementing appropriate strategies in difficult situations. Being able to access strategies outside of therapy in a range of situations is an important component that is necessary to facilitate therapeutic change. Thus, both active engagement in treatment and a good therapeutic alliance are the underpinnings of effective dialectical behavior therapy treatment. Engagement can be a challenge in dialectical behavior therapy treatment and several studies have noted dropout rates between 20 and 50% [9.10]. Dropout from treatment has been associated with a higher risk of self-injury and suicide within this population [17].

The integration of a mobile app into dialectical behavior therapy treatment is congruent with the goals and techniques of psychotherapy for borderline personality disorder. It is hypothesized that the integration of an app into dialectical behavior therapy treatment will facilitate the use of appropriate therapeutic techniques and reduce dropout. A recent study on dialectical behavior therapy found that using dialectical behavior therapy skills less was linked to higher dropout from treatment and that more frequent of use dialectical behavior therapy skills was associated with less self-harm [17].

Currently, there are only a few studies that have examined how mobile apps can be integrated into dialectical behavior therapy treatment for borderline personality disorders [18-21]. Results from these studies have indicated the potential feasibility of using apps in dialectical behavior therapy treatment, but these studies also concluded that further research was needed.

The aim of the study was to identify participant perspectives on dialectical behavior therapy treatment and the use of a mobile app as a tool to enhance and support this therapy. Specifically, the study was designed to capture the experiences of participants who used the app during their dialectical behavior therapy treatment and their perceptions of using the app to facilitate therapeutic goals in treatment.

## Methods

### Participants

Participants were recruited from two psychiatric outpatient clinics located in Region Zealand, Denmark. Participants were invited to participate in the study if they met the inclusion criteria of a primary diagnosis of personality disorder (ICD-10 definition) and being able to participate in group treatment that used a dialectical behavior therapy framework. Several groups were used in order to capture a range of therapist, patient, and group characteristics.

### Treatment

Dialectical behavior therapy [7] has a solid evidence base that demonstrates its effectiveness in treating personality disorders. The therapy was initially developed to help patients with self-injurious and suicidal behavior [7], but it has been adapted for broader clinical application (such as for treatment in emotional instability or substance abuse problems)[7].

Dialectical behavior therapy has its roots in cognitive behavior therapy and focuses on changing unhelpful behavioral patterns by acknowledging feelings, thoughts, and behaviors. The primary dialectical principle in the therapy is that one needs to accept circumstances while working toward change. Throughout dialectical behavior therapy, people develop a range of skills such as core mindfulness, distress tolerance, emotion regulation, and interpersonal effectiveness. These skills help the individual to identify and regulate their emotions, to increase their self-respect, to increase their self-efficacy, and to reduce behaviors such as self-injury [22].

Treatment in this study consisted of groups of 6 to 8 people participating in 20 2-hour group sessions each week. Group sessions combined psychoeducation, skills training, self-reflection, and group discussion. Participants also attended individual sessions with a therapist every fortnight in order to discuss issues and to review progress. An important part of treatment was recording mood and implementing skills (working with relationships, regulating emotions, and building resilience to cope with difficult situations) between weekly sessions.

### Mobile App

The mobile app was developed by a company with extensive experience in the design and implementation of e-health solutions in psychiatry (Monsenso A/S). This organization has conducted clinical trials using mobile apps for both bipolar disorder and depression [23]. The mobile app platform was adapted from earlier research trials. The app was to be used as an alternative to the traditional paper journal used in therapy.

**Figure 1:**
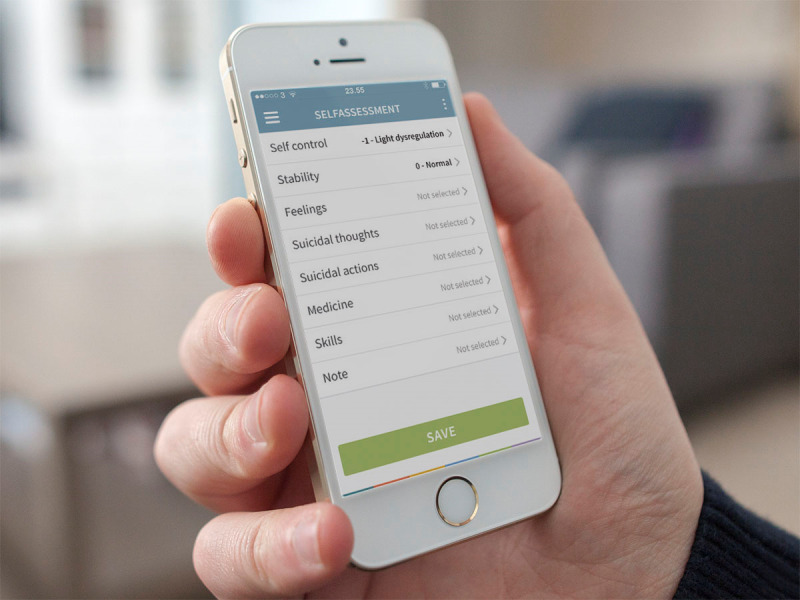
Self-assessment function menu of the mobile app.

**Figure 2:**
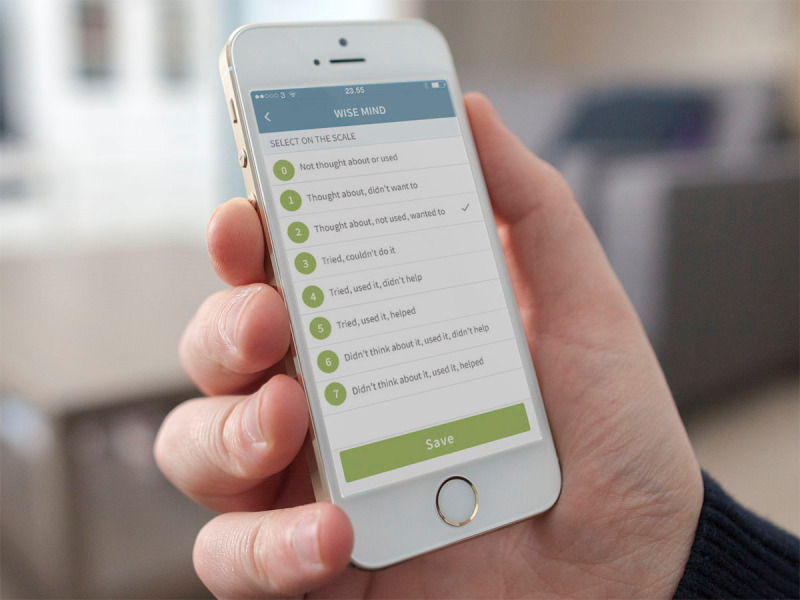
Dialectical Behavior Therapy folder function with strategies.

Key elements from dialectical behavior therapy treatment were adapted to the mobile app. These elements included psychoeducation, the recording and monitoring of mood, descriptions and implementation guidelines for a range of cognitive behavioral strategies to deal with challenging situations, and memos where a person could note their experiences *in situ*. The app contained a range of functions that could be accessed by the participant when he or she desired. A Self-Assessment function allowed participants to input information about their mood, behavior, sleep patterns, or any other parameter they wished to monitor (Figure 1). Data were summarized in graphical form and could be accessed using the Visualization function. The Early Warning Signs and Triggers functions allowed the participant to identify and note factors that preceded emotional reactions or inappropriate behaviors. These functions were often used in conjunction with the dialectical behavior therapy Folder function (Figure 2) which contained a range of strategies that could be used when challenges were experienced. The Medicine function allowed participants to create an overview of their medication and to set reminders if they had difficulty with adherence.

Common aspects of mobile apps such as reminders (push notifications) to complete daily monitoring were also incorporated into the app. App functions were accessible in all situations and the app was used as a support tool outside the therapy sessions.

Information that was input into the app by the participants was saved on a secure server that could be accessed by both the person and their therapist. Information that was input over several days or weeks could be summarized into graphs. These visual summaries could be used to provide an overview of symptom and mood fluctuations over time and to identify specific situations that had been difficult for them for discussion with the therapist in face-to-face appointments; information stored in the app facilitated recall of these situations during therapy sessions.

### Design and Evaluation

The study was an open clinical trial where all participants receiving dialectical behavior therapy group treatment were invited to participate. Participants using the app completed an evaluation questionnaire where they rated their satisfaction with dialectical behavior therapy treatment, alliance with therapist, perceived usefulness of the app, and usage of app between sessions. Responses were rated using a 10-point Likert scale ranging from 1-not at all helpful to 10-extremely helpful or useful.

Descriptive data regarding diagnosis, gender, age, and previous psychiatric treatments were collected. A subset of participants were asked to participate in semistructured interviews where they could describe their experiences using the app; a number of general topics were covered and content was determined by participants. An overview of the topics covered in the semistructured interview and evaluation questionnaire is presented in Textbox 1. Interviews lasted up to one hour, took place at the psychiatric clinic, and were recorded on an iPad. Written and verbal informed consent were obtained from each participant. Two researchers who were experienced psychologists and trained in the use of qualitative methods (SFA and CJP) were responsible for conducting the interviews.

**Topics covered in the semistructured interviews and in the evaluation questionnaire. DBT: dialectical behavior therapy.**

**Prompts and questions**

Describe your attitudes and use of technology in your daily life.

Describe your experiences in DBT treatment.

Describe your relationship with your therapist.

How (if) has the app been integrated into DBT treatment?

How (if) has the app influenced your relationship with your therapist?

What features of the app (if any) were useful in treatment?

What difficulties (if any) where encountered using the app in treatment?

What improvements (if any) could be made to the app?

Interviews were transcribed verbatim and analyzed using thematic analysis [24], which has been widely used for identifying, analyzing, and reporting patterns (themes) within qualitative data [25]. Two members of the research team (SFA and CJP) independently identified main themes in each interview and met afterward to reach consensus about main themes across all the interviews. Themes that were identified from each of the eight interviews were collapsed into main themes for each topic, and it was noted how many participants endorsed each of these themes. Quotes from participants that illustrate the themes were identified and selected from the interviews. Data from the evaluation questionnaire were summarized using descriptive statistics.

This study used a mixed methods approach, where data from questionnaires (quantitative) and interviews (qualitative) were collected at the same time and then integrated in a concurrent nested design [26]. An emphasis was placed on qualitative information generated from the interviews as this captured user experience more comprehensively, but quantitative data was used in order to contextualize the themes that were identified from the interviews.

### Ethical Considerations

The study was approved by the national association for the storage and analysis of health data (Datatilsynet j.nr. 2008-58-0020 16-000032, REG 135-2016). Written and verbal consent were obtained from all participants involved in the study. Participants were assigned a project number and all information was stored under that number to ensure confidentiality and anonymity.

### Data Management and Storage

Data were stored and handled as confidential information in accordance with Danish legislation for the protection of health data (Datatilsynet j.nr. 2008-58-0020 REG 135-2016). Electronic data were stored on a secure database administered by the Region Zealand Information and Technology department.

## Results

The study consisted of 4 dialectical behavior therapy treatment groups that consecutively trialed the app over a period of 1 year. All participants (N=24) in the treatment groups were asked to complete an evaluation questionnaire and a subset (n=8) were asked to participate in semistructured interviews. Most treatment group participants also participated in the app evaluation (n=20); the participants who did not complete the evaluation questionnaire either could not be contacted (n=2) or did not wish to participate in the evaluation (n=2). The majority of participants were female (female: 19/20, 95%; male: 1/20, 5%). Participants had a mean age of 28.9 (SD 6.7) years and 75% (15/20) had been diagnosed with borderline personality disorder. Table 1 provides a summary of the participant characteristics.

**Table 1:** Characteristics of the participants using the app in dialectical behavior therapy treatment.

Characteristics of the participants (n=20)		Value
Age (years), mean (SD)		28.9 (6.7)
**Gender, n (%)**		
	Female	19 (95)
	Male	1 (5)
**Psychiatric Diagnosis and ICD-10^a^, n (%)**		
	Borderline personality disorder (F60.31)	15 (75)
	Mixed personality disorder (DF61.0)	4 (20)
	Anxious personality disorder (DF60.6)	1 (5)
**Single or multiple diagnoses**		
	Single diagnosis	11 (55)
	Multiple psychiatric diagnoses (predominantly anxiety, depressive, or personality disorders).	9 (45)

^a^ISD-10: International Statistical Classification of Diseases, Tenth Revision.

Table 2 contains a summary of the responses from the evaluation questionnaire. The responses from the questionnaire indicated that participants used the app 20.3 (SD 6.3) weeks during treatment and indicated that participants perceived that treatment was helpful, the alliance with their therapist was strong, the app was helpful during dialectical behavior therapy treatment, and their use of the app between sessions was moderate.

**Table 2:** Participant responses to the evaluation questionnaire about the app.

Question content	Responses
Weeks using app in treatment (weeks)	20.3 (6.3)
How much has dialectical behavior therapy treatment helped (out of 10)^a^	7.4 (1.6)
How useful was the app was in treatment (out of 10)	7.2 (2.2)
How useful was the app in building an alliance with therapist (out of 10)	7.0 (2.3)
How much the app has been used between sessions (out of 10)	6.9 (2.4)

^a^Questions were rated on a Likert scale ranging from 1 = not helpful at all to 10 = extremely helpful.

The participants who were interviewed (n=8) were all female (8/8, 100%), most of the women had been diagnosed with borderline personality disorder (6/8, 75%), and more than half (5/8, 63%) had multiple psychiatric diagnoses (other personality disorders, anxiety disorders or ADHD). The majority of women were aged between 25-35 years old and all the women had been in contact with psychiatric services for at least 5 years. Thus, the women interviewed were considered to have complex mental health problems and had received a range of pharmacological and psychosocial interventions prior to participating in dialectical behavior therapy treatment.

Table 3 contains the results from thematic analysis of the qualitative interviews. The main themes that were identified for each topic and representative quotes from participants (quotes that capture the essence of each theme) are presented. The main themes were a positive perception of treatment and therapeutic alliance, and that the app facilitated the access and implementation of relevant dialectical behavior therapy techniques during treatment.

**Table 3:** Common themes and participant quotes from qualitative interviews.

Topics explored	Main themes (number of themes endorsed)	Participant quotes
Attitudes toward using technology	Participants were comfortable using technology in all aspects of daily life (7/8) Positive attitudes about using an app in treatment (5/8) Technology needs to be personalized to a person’s needs (3/8)	“It is good idea as I have my mobile with me all the time” (participant 2) “Using a phone is better than paper because it is easier to remember and register” (participant 4)
Experience of dialectical behavior therapy treatment	Positive experience (7/8) Treatment provided insight and skills to cope with difficulties (6/8)	“DBT^a^ was great, I have learned lots of skills to use in my daily life, I am more calm and I do not have such a bad temperament now” (Participant 4) “ I have learnt about myself and I found out why I act in the way I do and when I feel bad what I can do to make it better” (Participant 5)
Relationship with therapist	There was a good relationship where patients felt safe and understood (7/8)	“It (our relationship) has been really good, I have been to many psychologists where I wasn’t understood but I was told there is something wrong with me. But now there was someone who finally understood me and helped me develop another perspective of myself” (participant 4)
Experience of using the app in treatment	It was easy to access when needed (5/8)	“It was really good because you are focused in another way. You are more aware what can fool you and how you can use the skills you have learnt (participant 1) “As soon as discover I have used a skill, so could open the app and register it. I didn’t need to remember I had used that skill” (participant 7)
Useful parts of app in dialectical behavior therapy treatment	The app was useful in recording mood and use of skills (7/8) The app was useful in recording reflection (5/8)	“I have more awareness of the specific skills that can help me if I am in a situation that is difficult to cope with. If I had an argument with my girlfriend, I could take a break and read in the app what I need here so I don’t switch over to an old response” (participant 1) “I can register my mood, feelings and one has notes where you can write what you have done during the day and remember how you have used the skills and how your mood has been” (participant 6)
The use of the app and impact on relationship with therapist	The app provided a common base and promoted collaboration when examining and discussing problematic situations (6/8)	“The app allowed my therapist to follow how I am going and to provide support if required. It provided us with a common overview of what we can examine and discuss together” (participant 6) “The app helped me work better with better with my therapist as she could quickly get insight into how I have been and we had more time to discuss things that were important rather than I have to try and remember the all difficulties I have had” (participant 8)
Advantages of using the app in dialectical behavior therapy treatment	The functions of the app were easily accessible (8/8) It helped patients be more aware of treatment issues inside and outside of therapy (5/8) Patients could access skills when they needed them (5/8)	“I am more aware of the skills and it does not require so much effort to take out my mobile phone discretely and use them” (participant 6). “I can see what I have done hangs together with my feelings” (participant 3) “I am aware of what skills I used yesterday and what I should use today” (participant 4)
Challenges using the app in treatment	The app should function without major problems and IT^b^ support should be available (6/8) There are some technical challenges (2/8) and lacks some flexibility (3/8)	“The scroll function is irritating so it can take a long time to find the part of the app you want” (participant 3) “There are things on the app that are irrelevant” (participant 5) “It can be hard to add your own notes and find them again” (participant 6)

^a^DBT: dialectical behavior therapy.

^b^IT: information technology.

## Discussion

### Overview

The following study examined user perspectives of incorporating an app into dialectical behavior therapy treatment for personality disorders. Results from the qualitative interviews and quantitative responses indicated that the majority of participants were comfortable with using technology in their daily life, and that they perceived the app as a positive addition to psychotherapy. Dialectical behavior therapy treatment was perceived as a positive experience, and many participants highlighted that they had a good alliance with their treatment provider. Many participants described feeling safe and understood by their therapist. Thus, the context in which the evaluation of the app took place was an environment where participants had a positive attitude toward psychotherapy and technology.

### Ease of Access and Facilitating Psychotherapy

Participant perception of the app based on in-depth interviews was predominantly positive, and they highlighted how the app was a useful tool in recording mood or behavior and for accessing relevant skills to help with difficulties. There was a clear perception that the app facilitated access to many aspects of dialectical behavior therapy both in session and in the real world.

Access to the app was facilitated by the fact that participants always had their phone with them, and that using one’s mobile phone in public is perceived as normal. Furthermore, accessing material on the app did not require a change in behavior or require them to remember to take something extra (for example, a manual or notebook). Thus, the incorporation of dialectical behavior therapy material on the mobile phone was built upon existing habits.

The quantitative data from the questionnaires supported this theme of accessibility to relevant information; 75% (14/20) rated the app as very useful in dialectical behavior therapy treatment. Interestingly, while the app was rated as useful, how often (the frequency) participants accessed the app varied greatly. Over half of the participants (12/20, 60%) stated that they accessed the app a lot, but 15% (3/20) stated that they accessed it sometimes, and 25% (5/20) responded that they accessed it a little or not at all. People who did not access the app often between sessions gave a variety of reasons such as they did not have the energy to use the app, they could not see the relevance, or they simply forgot to use it.

This considerable variation in usage despite a positive perception of its usefulness highlights the importance of identifying factors that facilitate or hinder engagement with the app. This issue is particularly important given that engagement in treatment is often linked to effectiveness. A recent review of cognitive behavior therapy interventions using mobile apps also found that despite positive ratings of the usefulness of an app, the ratings did not necessarily reflect longitudinal engagement [24].

### Promotion of Awareness and Reflection

A second prominent theme that was identified from the interviews was that the app helped to increase awareness and to facilitate reflection. This learning and reflection were reinforced during individual sessions where therapists and users would discuss the graphs generated by self-monitoring, and outside the session where the use of push notifications (reminders that were sent via mobile phone) helped users to remember to record their mood and activities. Regularly recording mood and reviewing the results generated by the app with the therapist was perceived as useful and to support the core components of dialectical behavior therapy treatment.

This theme of the app being a useful tool in therapy was supported by the quantitative data where 70% (14/20) of participants rated the app as very to extremely useful in treatment and only one person rated the app as not useful at all.

### Building Therapeutic Alliance

None of the participants perceived the app as having a negative impact on the therapeutic relationship, in contrast to the concern that technology may disrupt the therapeutic process. A key theme identified from the interviews was that the use of the app was actually perceived to promote better collaboration between therapist and patient as users stated that it provided a common source of information which could be used for in-depth discussion.

Results from the questionnaires also supported the theme of strengthening therapeutic alliance; 80% (16/20) felt it contributed to building a good therapeutic alliance and only 10% (2/20) of participants felt it contributed very little or nothing at all to the therapeutic relationship.

Furthermore, dropout rates from this intervention were under 10% which is significantly less than what is expected for dialectical behavior therapy treatment within this population. While the study design prohibits the conclusion that integration of the app into dialectical behavior therapy treatment reduces dropout rates, it does not appear that integration of an app into dialectical behavior therapy treatment was associated with higher dropout rates.

### Technical Problems Can Result in Disengagement

Overall, participants were accepting of and comfortable with integrating this mobile technology in their therapy and relatively few disadvantages were identified. One participant highlighted a technical problem regarding the app where it would freeze when updating. This technical problem resulted in that participant not using the app until the problem was rectified. Several other participants commented on the importance of the app functioning reliably and smoothly, and that technical support should be readily available to quickly solve any problems that arise.

The app functions that were most used by participants were self-assessment, visualization, and the therapy folder with strategies. General factors that influenced the frequency of use of different functions were the perceived relevance of the function, active use of the function during individual sessions, and ease of use. Interestingly, a number of people noted their desire to use the app varied depending on their mood where low mood was associated with reduced use. A couple of people commented that their motivation to use the app deceased over time as they became familiar with its content.

None of the participants who were interviewed identified any negative or harmful effects of using the app, but one person did have general concerns about other people potentially accessing or using personal data from the app. Overall, participants were confident in the security of the data stored in the app, but they were concerned that information could be accessed by people other than those to whom they had given specific permission to (such as the treating clinician). This perception of data security was as a consequence of the highly personal nature of some of the reflections stored in the memo function.

### Personalizing Functions May Increase Engagement

There were several suggestions to improve the app; the most common was adaptation of the scroll function so the app could remember where the user was when last accessing or registering information. Another suggestion was to increase the flexibility of the app by being able to personalize different functions (for example, to be able to switch off some functions and to expand others for streamlined access to the most frequently used functions).

Finally, a subgroup of participants who had previously used the written dialectical behavior therapy workbook (n=6) were asked to reflect about using the app compared to the written manual. The majority of this group (5/6, 83%) preferred using the app over using the paper manual; they highlighted advantages of the app such as portability, ease of access, and less chance of losing information or worksheets. One participant suggested that having both electronic and paper formats could be helpful depending on the context. For example, this person preferred using the paper manual when at home so they had access detailed information and preferred using the app when not at home as it was more portable and convenient.

### Participant Engagement is Only Half the Picture in Successful Implementation

While this study focused on user perspectives, it is important to acknowledge the system in which the app was implemented. The app was seen as a support tool for psychotherapy, and therefore, the clinical staff also played an important role in the integration of the mobile app into treatment. Staff were responsible for introducing and teaching participants about app functions, following up on data input by the participants, and incorporating output from the app (for example, graphs, summaries, and notes from participants) into therapy sessions. Focus group interviews with clinical staff were conducted to capture their perspectives as another part of the evaluation. Staff highlighted the importance of receiving adequate training and ongoing support throughout treatment so that they were comfortable using the app with their clients. Furthermore, staff stated there was the need for sufficient time to learn and integrate the app meaningfully into clinical treatment. This meant personnel needed to have a reduced caseload while learning and integrating the app, thus support and approval from their line managers was required.

### Study Limitations

The study had a number of limitations. First, results were based on a small sample of participants which is a common feature of qualitative studies. As a result, it may be difficult to generalize the findings to all people who receive dialectical behavior therapy interventions for personality disorders. Second, the study focuses on participant perception of the experience of using the app; therefore, findings may not reflect actual behavior. Future studies should incorporate sensor data and daily monitoring information from the app to quantify actual usage. This function was not available in this version of the app. Finally, no information regarding symptoms, behavioral change (for example, self-harm), or measurement of therapeutic alliance was systematically collected in this study. Thus, it was not possible to determine if using the app affected dialectical behavior therapy treatment effectiveness. Future studies could determine the efficacy of integrating an app into dialectical behavior therapy using suitable study design.

### Conclusions

Our study investigated participant experience of the integration of a mobile app into dialectical behavior therapy treatment for personality disorders. Participants had a positive attitude toward the use of technology and perceived dialectical behavior therapy treatment as useful in treating their mental health difficulties.

Qualitative and quantitative results indicated that the app was widely accepted and used in treatment by participants. Participants described that the content of the app was easily accessible and often helped to facilitate self-monitoring and the use of strategies and reflection techniques that were learned in therapy sessions in the real world. While the app was seen as a useful addition to treatment, usage of the app between sessions varied indicating that engagement fluctuated. Given the link between engagement and positive outcomes, further research into factors that affect engagement with technology is needed. There were relatively few disadvantages identified by users although there were some suggestions to improve the design of the app to increase its flexibility; this suggests personalized app functions may warrant further exploration. The positive findings from this study are in line with a recent review [27] that highlighted the feasibility and acceptability of integrating technology into clinical treatments for serious mental illness.

It is important to acknowledge that this evaluation does not provide information on whether the integration of an app into dialectical behavior therapy treatment had a positive effect on treatment outcomes. The results suggest that an app could have benefits and that further evaluation is warranted. Researchers and clinicians should continue to collaborate with psychiatric service users in to understand how technology can be meaningfully integrated into psychotherapy in order to promote engagement and to improve treatment outcomes.

## Abbreviations

DBT: dialectical behavior therapy
ICD-10: International Statistical Classification of Diseases, Tenth Revision
IT: information technology
